# Synergistic Modulation of the Gut–Brain–Immune Axis by a Dual *Lactobacillus* Combination in a Murine IBS Model

**DOI:** 10.4014/jmb.2507.07018

**Published:** 2025-10-27

**Authors:** Lim Song, Hanbyeol Lee, Joon-Sun Choi, Seoyeon Park, Hyein Jeong, GwangPyo Ko

**Affiliations:** 1 Department of Environmental Health Sciences, Graduate School of Public Health, Seoul National University, Seoul 08826, Republic of Korea; 2 Center for Human and Environmental Microbiome, Seoul National University, Seoul 08826, Republic of Korea; 3 Bio-MAX/N-Bio, Seoul National University, Seoul, Republic of Korea; 4 KoBioLabs Inc., Seoul, Republic of Korea

**Keywords:** Irritable bowel syndrome (IBS), gut microbiota–immune–brain axis, *Lactobacillus* spp., zymosan-induced mouse model, anxiety-like behavior

## Abstract

The gut microbiota is a key regulator of immune and neuroendocrine pathways along the gut–brain axis. Disruption of this bidirectional communication contributes to irritable bowel syndrome (IBS), a multifactorial disorder associated with gastrointestinal dysfunction and psychiatric comorbidities. Although microbiota-targeted therapies are promising, most current studies rely on single-strain interventions with limited efficacy, and the bioactive components as well as their host-mediated mechanisms remain insufficiently characterized. Here, we demonstrate that oral co-administration of *Lactobacillus paracasei* KBL382 and *Lactobacillus plantarum* KBL396 synergistically ameliorates IBS-like symptoms in a zymosan-induced mouse model. The combination therapy outperformed individual strains in reducing colonic shortening, abnormal cecal morphology, mucosal inflammation, and anxiety-like behaviors. These effects were accompanied by bidirectional neurobiological changes, including downregulation of colonic brain derived neurotrophic factor (BDNF) and serotonin 3A (5-HT3A), and restoration of hippocampal serotonergic signaling. Immunologically, the treatment decreased pro-inflammatory M1 macrophages and inflammatory dendritic cells (DCs), while increasing tolerogenic DCs and regulatory T cells in mesenteric lymph nodes. Furthermore, >100 kDa macromolecular fractions isolated from both strains enhanced the IL-10/IL-6 ratio and serotonin transporter (SERT) expression in vitro. These effects were abolished by protease or mutanolysin treatment, implicating structurally integrated peptidoglycan–protein complexes as key immunoregulatory and neuroactive components. The complexes engaged MyD88-dependent signaling pathways, promoting regulatory immune phenotypes. Importantly, therapeutic effects were preserved in microbiota-depleted mice, demonstrating a microbiota-independent, host-targeted mechanism. These findings demonstrate that defined *Lactobacillus* strains synergistically modulate neuroimmune pathways via bioactive macromolecules, offering a host-directed strategy for managing the multifactorial symptoms of IBS.

## Introduction

Irritable bowel syndrome (IBS) is one of the most prevalent functional gastrointestinal disorders, characterized by abdominal pain and altered bowel habits such as bloating and stool irregularities [1]. It significantly reduces quality of life and contributes to substantial healthcare costs [2]. Despite its high prevalence, the pathophysiology of IBS remains incompletely understood due to its heterogeneity and multifactorial nature, with no identifiable structural abnormalities, making diagnosis and treatment difficult [3, 4].

IBS is increasingly recognized as a disorder of the gut-brain axis, often accompanied by psychological comorbidities such as anxiety and depression [5]. Dysregulation of neuroendocrine signaling and immune responses along this axis is thought to contribute to symptom persistence [6-8]. Among key mediators, serotonin (5-HT) plays a central role by regulating both gastrointestinal motility and visceral sensitivity, as well as emotional processing [9]. Secreted primarily by enterochromaffin (EC) cells in the gut, serotonin activates 5-HT3 receptors on afferent neurons, transmitting nociceptive signals to the central nervous system [10, 11]. Clinical studies have reported increased 5-HT levels and reduced serotonin transporter (SERT) expression in IBS patients, potentially amplifying pain and altering bowel function [12, 13]. This dysregulation of serotonergic signaling, particularly via the 5-HT3 pathway, contributes to visceral hypersensitivity, which is a hallmark of IBS, and supports the use of 5-HT3 antagonists in symptom management, especially in diarrhea-predominant subtypes [14, 15].

In addition to serotonin, brain-derived neurotrophic factor (BDNF) has been implicated in IBS-related pain. Elevated BDNF expression in colonic mucosa correlates with heightened visceral sensitivity, and its inhibition has been shown to reduce pain perception in both clinical and experimental settings [16, 17]. These findings highlight the role of neuroimmune crosstalk in IBS pathophysiology.

Another key feature of IBS is low-grade inflammation and impaired mucosal barrier function, which are characterized by increased infiltration of mast cells and lymphocytes and elevated levels of pro-inflammatory cytokines such as IL-6, IL-8, and TNF-α, known to sensitize enteric neurons and exacerbate visceral pain [18]. The gut microbiota has emerged as a central regulator of these immune disturbances, given its established role in maintaining epithelial integrity, immune homeostasis, and neuroendocrine signaling within the gut-brain axis [19, 20]. Dysbiosis is frequently observed in IBS patients and has been linked to both barrier dysfunction and immune activation. While reduced abundance of beneficial genera such as *Bifidobacterium* is consistently reported, changes in *Lactobacillus* appear to be more variable, with studies documenting both increases and decreases across different IBS populations [21-23].

Given this, microbiota-targeted therapies such as probiotics have garnered attention for their ability to restore microbial balance and modulate host immunity. Clinical trials have reported symptomatic improvements following probiotic supplementation, including enhanced barrier integrity and reduced inflammation in IBS patients. However, most microbiota-based studies in IBS to date have relied on single-strain probiotic interventions, which limit the scope of host modulation and often yield inconsistent outcomes. Given the multifactorial nature of IBS, encompassing both immune dysregulation and neuropsychological comorbidities, a multi-strain approach holds greater therapeutic promise by targeting multiple pathological axes simultaneously.

In this study, we investigated the therapeutic potential of a defined combination of *Lactobacillus paracasei* KBL382 and *Lactobacillus plantarum* KBL396, selected based on their complementary functional properties. Our research group has previously demonstrated the anti-inflammatory efficacy of *L. paracasei* KBL382 in murine models of atopic dermatitis and colitis [24, 25], while *L. plantarum* KBL396 exhibited effects on stress response and neuroimmune function in preclinical settings and is patented for neuroprotective applications in the treatment of neurological disorders. These prior findings provided a strong biological rationale for evaluating their combined effects in the context of IBS, where immune and behavioral dimensions are closely interlinked.

We hypothesized that co-administration of these two strains would result in synergistic therapeutic effects, surpassing the efficacy of each strain alone by orchestrating immune and neurobiological regulation along the gut–brain axis. Furthermore, to gain mechanistic insight into the cellular and molecular underpinnings of this effect, we aimed to identify microbial-derived bioactive compounds and elucidate their contributions to host immune signaling. Characterizing strain-specific functional molecules and their impact on host neuroimmune pathways is essential for the development of next-generation precision microbiome therapeutics. In summary, this study presents a synergistic probiotic strategy with mechanistic relevance to the gut–immune–brain axis, offering a promising microbiota-based intervention for IBS.

## Materials and Methods

### Animals

All animal experiments were conducted in accordance with institutional guidelines and were approved by the Institutional Animal Care and Use Committee (IACUC) of Seoul National University, Korea (Approval No. SNU-190110-2-1). Seven-week-old male specific pathogen-free (SPF) C57BL/6 mice used in this study were purchased from Central Lab Animals Incorporated (Republic of Korea). Mice were housed under specific pathogen-free (SPF) conditions with a controlled 12 h light/dark cycle (lights on at 7:00 a.m. and off at 7:00 p.m.), ambient temperature maintained at 25 ± 2°C, and relative humidity of 45–65%. Sterilized food and water were provided ad libitum. Sixty-six mice were acclimated for 1 week before the test then randomly assigned into 6 experimental groups (11 mice per group in 3 cages).

### Bacteria Preparation

*L. paracasei* KBL382 (Accession No. KCTC13509BP) and *L. plantarum* KBL396 (Accession No. KCTC13278BP) were isolated from fecal samples of healthy Korean adults and stored at −80°C until use. Lyophilized bacterial preparations were administered to mice by oral gavage at a total dose of 4 × 10^9^ CFU/day. For single-strain groups, mice received either *L. paracasei* KBL382 or *L. plantarum* KBL396 at 4 × 10^9^ CFU/day. For the mixture group, the dose was equally divided, with 2 × 10^9^ CFU/day of each strain, maintaining the same total inoculum of 4 × 10^9^ CFU/day across all groups. Administration was performed once daily, starting 7 days prior to zymosan injection and continued until the experimental endpoint. This regimen was chosen based on prior work and published studies showing that a dose of ~10^9^ CFU/day is effective in modulating gut inflammation and visceral hypersensitivity, and that initiating probiotic treatment one week before the inflammatory challenge allows sufficient time for colonization and host adaptation [24-27]. For in vitro studies, both live and pasteurized bacterial preparations were generated and quantified. Details of bacterial culture, viability assessment, and pasteurization procedures are provided in the Supplementary methods.

### Zymosan Induced IBS Animal Model

The zymosan-induced IBS model was chosen because it reliably induces visceral hypersensitivity and low-grade mucosal inflammation without overt tissue damage, thereby closely resembling the pathophysiological features of human IBS, as reported in previous studies [28-31]. IBS-like symptoms were induced by intracolonic administration of zymosan for three consecutive days. To induce IBS-like symptoms, a volume of 0.1 ml zymosan suspension (30 mg/ml in PBS; Sigma-Aldrich, USA) was administered into the colons using a 22-gauge long stainless-steel feeding needle [28, 29]. Mice were anesthetized by isoflurane inhalation during the performance. Either vehicle or zymosan was given daily for 3 consecutive days (days 1, 2, and 3). Naïve group was subjected to the same procedure as those in zymosan-induced control group except intracolonic injection with 0.1 ml PBS. Three experimental groups were pretreated with either single or combined *Lactobacillus* strains for seven days prior to zymosan injection, while control groups received PBS orally. Mice were randomly assigned to six groups (*n* = 11 per group): (1) oral PBS + intracolonic PBS, (2) oral PBS + intracolonic zymosan, (3) oral amitriptyline (AMT; 30 mg/kg) + intracolonic zymosan, (4) oral *L. plantarum* KBL396 + intracolonic zymosan, (5) oral *L. paracasei* KBL382 + intracolonic zymosan, and (6) oral combination of both *Lactobacillus* strains + intracolonic zymosan. An additional cohort was pretreated with a broad-spectrum antibiotic cocktail (1 g/l ampicillin, 1 g/l metronidazole, 1 g/l neomycin, and 0.5 g/l vancomycin) in drinking water for one week to deplete resident microbiota, followed by daily administration of the *Lactobacillus* strains for seven days prior to zymosan injection. In AMT groups, drug treatment commenced on the first day of zymosan injection. This antibiotics regimen has been extensively validated in previous studies as an effective approach to induce gut microbiota depletion and dysbiosis, consistently demonstrating marked reductions in microbial diversity, distinct alterations in microbial composition, and associated changes in host metabolic and immune responses [33-35].

### Cell Isolation and Culture

Spleen cells were isolated from mice as follows: the spleen was aseptically resected, placed in ice-cold Roswell Park Memorial Institute 1640 (RPMI 1640) medium (Gibco, USA), and mechanically dissociated using a cell strainer. The collected splenocytes were centrifuged at 350 ×*g* for 5 min at 4°C, resuspended in ammonium-chloride-potassium (ACK) lysis buffer to remove red blood cells, and centrifuged again. The resulting cell pellet was resuspended in RPMI 1640 medium supplemented with 10% fetal bovine serum (FBS) and 1% penicillin-streptomycin to a final concentration of 1 × 10^6^ cells/50 μl and seeded into a 96-well round-bottom cell culture plate.Caco-2 human intestinal epithelial cells were maintained at 37°C in minimal essential Eagle's medium (MEM, Sigma) supplemented with 10% FBS (Life Technologies, Thermo Fisher Scientific, USA), 1% MEM nonessential amino acids (Sigma-Aldrich, USA), 100 U/ml penicillin, and 100 μg/ml streptomycin under a 5% CO_2_ humidified atmosphere.

For TLR2 blockade experiments, mouse splenocytes were isolated and cultured as described above. Cells were stimulated with LPS (100 ng/ml, Sigma-Aldrich) in the presence or absence of a selective TLR2 inhibitor (TL2-C29, InvivoGen, USA). The inhibitor was added at a final concentration of 50 μM and preincubated for 3 h before treatment with >100 kDa fractions.

### Bacterial Fractionation

Pasteurized *Lactobacillus* strains were sonicated and fractionated by molecular weight to isolate bioactive components. Enzymatic treatments were applied to selectively degrade proteins, DNA, RNA, and peptidoglycan. Detailed protocols for fraction preparation, size separation, and enzymatic degradation are described in the Supplementary methods.

### Transcript Analysis

Total RNA was extracted from mouse tissues, Caco-2 cells, and splenocytes, followed by cDNA synthesis and quantitative PCR. Gene expression levels were normalized to GAPDH and calculated by relative quantity (2–ΔΔCT) method. Details of RNA preparation, primer sequences, and qPCR conditions are described in the Supplementary methods.

### Enzyme-Linked Immunosorbent assay (ELISA)

After collecting blood samples, prefrontal cortex and hippocampus, and colon were collected and stored at –80°C until use. Brain tissues and mucosal samples were separately homogenized in RIPA buffer (1:10 w/v, Sigma-Aldrich) containing protease inhibitors. The protein concentration of each sample was determined using PierceTM BCA Protein assay kit (Thermo Fisher Scientific, USA). All samples were assayed in duplicate. The protein levels of BDNF were measured using a commercially available ELISA kit (R&D, USA) according to the manufacturer’s protocols. Blood was obtained and kept at room temperature for 30 min for clotting. Clotted samples were centrifuged at 1,800 rpm for 5 min at 4°C, and then divided in aliquots and immediately stored at –80°C until use. Concentration of protein of each sample were determined using PierceTM BCA Protein assay kit (Thermo Fisher Scientific) and Serotonin Ultrasensitive ELISA kit (Eagle Biosciences, USA).

### Behavioral Testing

Two classical behavior tests of anxiety, the open-field (OF) and elevated plus maze (EPM), were performed and automatically analyzed using a video tracking system (SMART 3.0 PanLab software, USA). Before starting, mice were habituated to the procedure room for 30 min. During all trials, two experimenters and the computer were posited behind a curtain to minimize disturbance. The testing area was illuminated with dim, indirect room light, and the same conditions were maintained for both tests.

Open-field test: The open-field arena consisted of a white polystyrene box (44 cm × 44 cm × 60 cm) with enclosed walls. A multiple-unit open-field maze with four activity arenas was used, allowing simultaneous analysis of four mice. To eliminate olfactory cues, each arena was cleaned with 95% ethanol and allowed to dry before introducing the next animal. Each mouse was placed in the center of the arena, and the test was run for 5 min. The following parameters were analyzed using the video tracking system: (1) total distance traveled, (2) distance traveled in the central zone, (3) time spent in the central zone versus total time, and (4) number of entries into the central zone.

Elevated plus maze: The EPM consisted of two open arms and two closed arms arranged in a cross shape, elevated 50 cm above the floor. At the start of each test, mice were placed at the center facing an open arm and were allowed to explore freely for 5 min. The frequency and time spent in open versus closed arms were recorded. The apparatus was cleaned with 95% ethanol between trials.

### Flow Cytometry

**Single cell preparation.** Dissected samples of mesenteric lymph nodes (mLNs) were immediately placed in a 5 ml conical tube containing cold Roswell Park Memorial Institute 1640 medium (RPMI 1640, Gibco) on ice. Single-cell suspensions were directly obtained via gentle manual grinding with the flat head of a syringe plunger on a 70 μm cell strainer. The collected cells were centrifuged at 350 ×*g* for 5 min at 4°C, then resuspended in ACK lysis buffer to remove red blood cells and centrifuged again. The resulting cell pellet was resuspended in RPMI 1640 medium and was filtered through a 40 μm cell strainer. After centrifugation (350 ×*g*, 5 min, 4°C), viable cells were counted using a hemocytometer with trypan blue counterstain. The cells were resuspended with RPMI medium containing 10% FBS, 1% penicillin-streptomycin to a concentration of 1 × 10^6^ cells/100 μl, and seeded into a 96-well cell culture plate. The cells were then incubated with 50 ng/ml Phorbol 12-myristate 13 acetate (PMA, Sigma Aldrich), 1 μg/ml Ionomycin (Sigma Aldrich), 4 μl/3 ml BD GolgiStop, and 1 μl/ml BD GolgiPlug (BD Biosciences, USA) for 4 hours at 37°C and 5% CO_2_.

**Staining of single-cell suspensions.** Cells were washed using 1 X PBS prior to addition of viability dyes (Zombie Aqua Fixable Viability Dye, USA) according to manufacturers’ instructions. Samples were incubated with anti-CD16/anti-CD32 blocking antibodies (USA) for 15min at 4°C. Subsequently, cells were stained with the following antibodies in FACS buffer for 30min at 4°C: anti-CD45-APC-Cy7 (BioLegend, 109824), anti-CD11c-FITC (BioLegend, 117306), anti-MHCII-BV421 (BioLegend, 107631), anti-CD11b-PE-Cy7 (BioLegend,101215), anti-F4/80-PE (BioLegend, 123110), anti-CD3-BV421 (BioLegend, 100228), anti-CD4-FITC (BioLegend, 100405), and anti-CD8-PE-Cy7 (BioLegend,100722), anti-CD103-APC (BioLegend, 121414), anti-CD25-APC (BioLegend, 102011), anti-IL-10-PE (BD Pharmingen, 561060), anti-IFN-r-APC (BioLegend, 505810), anti-FoxP3-PE (BioLegend, 126403). Cells stained for extracellular markers were fixed using the Cytofix(BD Biosciences). Intracellular cytokine staining was performed using Cytofix/Cytoperm (BD) according to the manufacturer’s instructions. Transcription factor staining was performed using Transcription Factor Buffer Set (eBioscience, USA). The results were collected using a FACS Canto II (BD Biosciences) and analyzed using FlowJo (BD Biosciences)

### Statistical Analysis

All data are presented as means ± standard error of the mean (SEM). Statistical analyses and graphing were performed using GraphPad Prism 10 (GraphPad Software, USA). Unless otherwise specified, geometric means and 95% confidence intervals were used to describe central tendency. Group comparisons were assessed using one-tailed Mann–Whitney U tests or one- and two-way ANOVA with Tukey's multiple comparison tests. as appropriate. A one-tailed Mann–Whitney U test was applied in cases where a priori hypotheses predicted a unidirectional change, thereby increasing test sensitivity under directional expectations. Differences were considered statistically significant at *P* < 0.05.

### Ethics Statement

All animal experiments were conducted in accordance with institutional guidelines and were approved by the Institutional Animal Care and Use Committee (IACUC) of Seoul National University, Korea (Approval No. SNU-190110-2).

## Results

### Enhanced Therapeutic Efficacy of Combined *Lactobacillus* Treatment in a Zymosan-Induced Mouse IBS Model

**Fig. 1:**
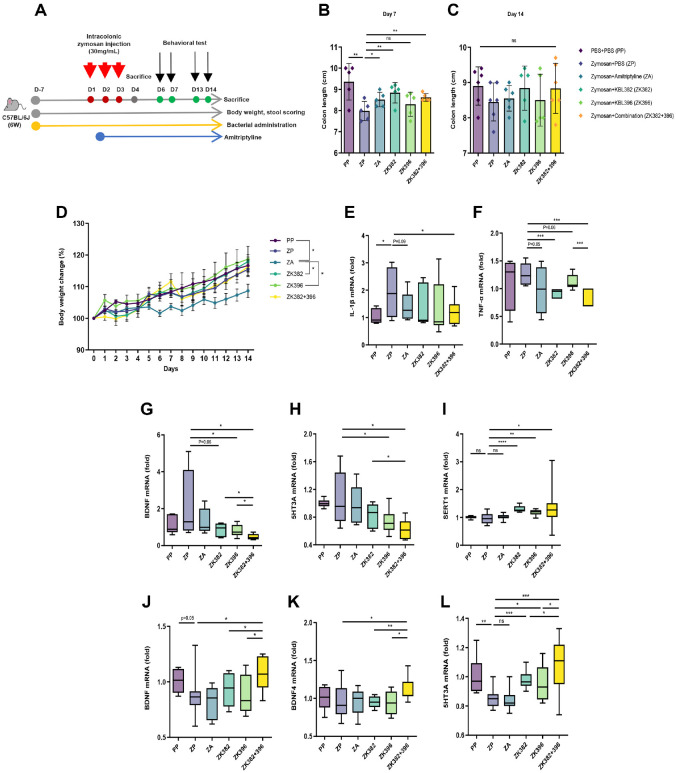
Combined treatment enhances therapeutic efficacy in a zymosan-induced murine model of IBS. (**A**) Schematic representation of the zymosan-induced IBS model in SPF mice for in vivo studies. Oral administration was started 7 days prior to zymosan injection. Intracolonic injection of zymosan suspension was performed over three consecutive days. (**B** and **C**) Colon length measured at the time of euthanasia on day 7 and 14. (**D**) Changes in body weight over the course of the experiment. (**E** to **I**) Quantification of IL-1β (**E**), TNF-α (**F**), BDNF (**G**), 5HT3A (**H**), and SERT1 (**I**) expression in colon tissue on day 7 by real-time qPCR. (**J** to **L**) Quantification of BDNF (**J**), BDNF4 (**K**), and 5HT3A (**L**) expression in brain tissue on day 14 by real-time qPCR. Expression levels were normalized to GAPDH expression. Symbols in (**B** and **C**) represent individual mice. Data in (**D**) are presented as mean±SEM. The box and horizontal bar in (**E** to **L**) represent the interquartile range and median of the correlation coefficients, respectively, while whiskers indicate the most extreme data points. Statistical analysis was performed using Mann-Whitney U-test and Two-way ANOVA with Tukey's multiple comparison tests. *P*-values are indicated as follows: *, *P* < 0.05; **, *P* < 0.01; ***, *P* < 0.001; ****, *P* < 0.0001; n.s. not significant. PBS, Phosphate-buffered saline; AMT, amitriptyline; PP, mice receiving intracolonic injection of PBS and oral administration of PBS; ZP, mice receiving intracolonic injection of zymosan and oral administration of PBS; ZA, mice receiving intracolonic injection of zymosan and oral administration of amitriptyline; ZK382, mice receiving intracolonic injection of zymosan and oral administration of *L. paracasei* KBL382; ZK396, mice receiving intracolonic injection of zymosan and oral administration of *L. plantarum* KBL396; ZK382+396, mice receiving intracolonic injection of zymosan and oral administration of *L. paracasei* KBL382 and *L. plantarum* KBL396 in combination.

The zymosan-induced IBS mouse model, which mimics diarrhea-predominant IBS, is characterized by visceral hypersensitivity, diarrhea, anxiety-like behaviors, weight loss, and mild systemic toxicity [28-31]. To evaluate the potential synergy of two *Lactobacillus* strains, we pre-administered *L. paracasei* KBL382 and *L. plantarum* KBL396, individually or in combination, for seven days prior to zymosan injection. Zymosan was then administered intracolonically for three consecutive days. Amitriptyline (AMT), a clinically used IBS treatment, served as a positive control (Fig. 1A).

On day 7, colon length was significantly improved in mice treated with *L. paracasei* KBL382, either alone or in combination, compared to the zymosan-only group (Fig. 1B). By day 14, differences among groups were no longer significant, likely due to natural recovery following cessation of zymosan stimulation, although early trends persisted (Fig. 1C). In contrast, AMT treatment caused dose-dependent reductions in body weight (Fig. 1D), underscoring the better safety profile of the probiotic treatment.

To assess epithelial barrier integrity, we measured occludin expression after TNF-α stimulation. The combination treatment significantly restored occludin levels, surpassing individual strain effects (Fig. S1A), suggesting enhanced barrier-preserving capacity. Additionally, only the combination group significantly reduced IL-1β and TNF-α expression in the colon (Fig. 1E and 1F), indicating superior anti-inflammatory efficacy. The same group also showed marked downregulation of BDNF and 5-HT3A, key markers of neuronal excitability and visceral hypersensitivity, while upregulating SERT expression, which is typically reduced in IBS patients (Fig. 1G-1I).

Given the involvement of the gut-brain axis in IBS, we further examined neurobiological markers in the brain. On day 14, BDNF and serotonin receptor levels were significantly elevated in the brains of *Lactobacillus*-treated mice, especially in the combination group. These increases were most prominent in the hippocampus, a region involved in emotional regulation (Fig. 1J-1L).

These findings indicate that *L. paracasei* and *L. plantarum* exert bidirectional regulation of key biomarkers in the colon and brain. In the colon, they reduce BDNF and 5-HT3A to alleviate IBS symptoms, while in the brain, they increase these markers to improve mood-related outcomes. The combination treatment amplified these effects, demonstrating a synergistic dual action on gut and brain dysfunction.

### Combined *Lactobacillus* Treatment Attenuates Anxiety-Like Behavior in a Zymosan-Induced Mouse Model

**Fig. 2:**
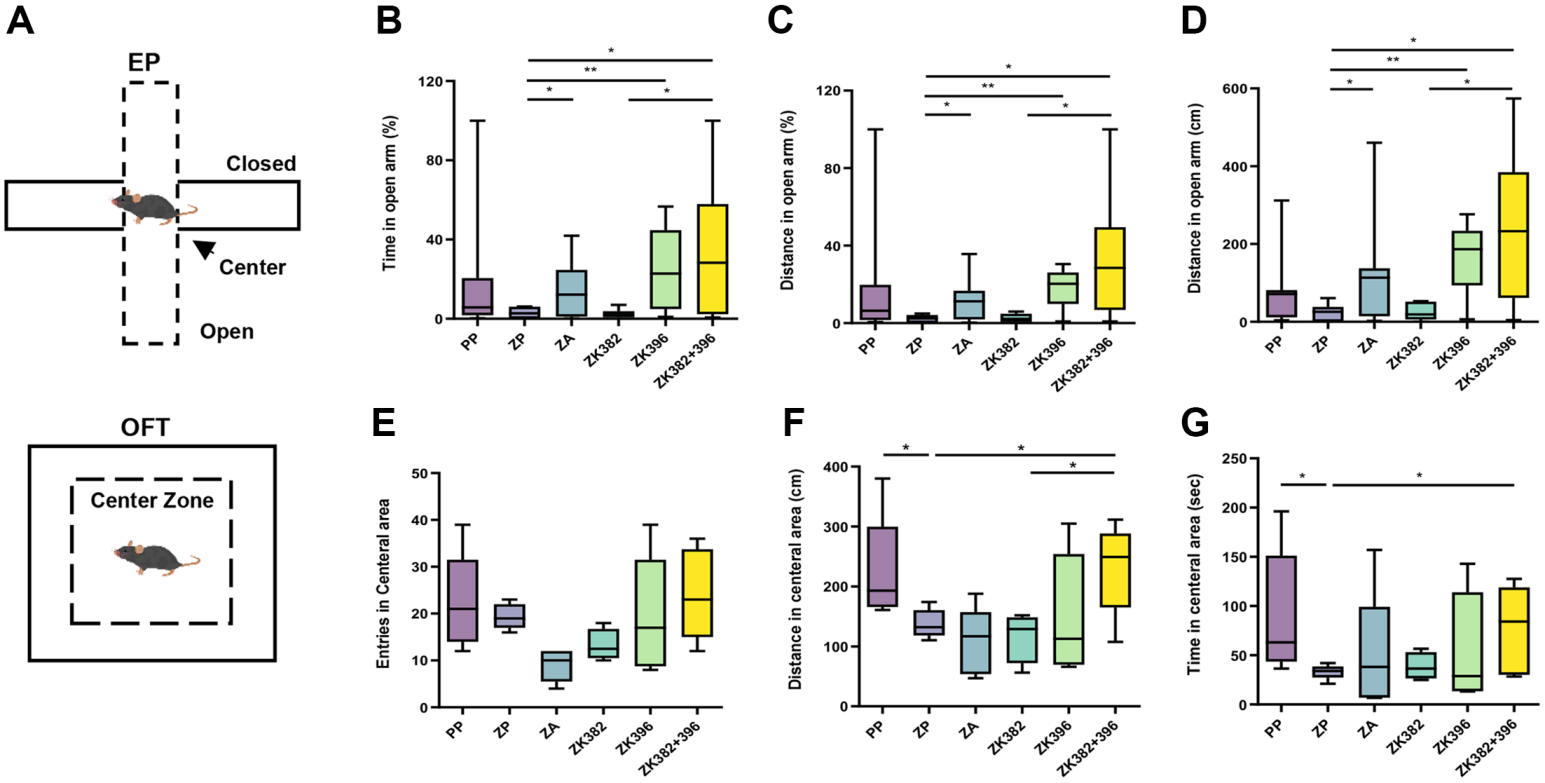
Combined administration alleviates anxiety-like behavior induced by intracolonic injection of zymosan. (**A**) Schematic diagram of the elevated plus maze (EPM) and open field test (OFT). (**B** to **D**) Summarized data of distance travel (**B** and **C**) and time spent (**D**) in the open arm of the EPM for the indicated groups on day 13. (**E** to **G**) Summarized data of entries (**E**), distance travel (**F**), and time spent (**G**) in the central area of the OFT from the indicated groups on day 14. Statistical analysis was performed using Mann-Whitney U-test. *P*-values are indicated as follows: *, *P* < 0.05; **, *P* < 0.01; ***, *P* < 0.001; ****, *P* < 0.0001; n.s. not significant. EPM, elevated plus maze; OFT, open field test.

To determine whether the biomolecular changes induced by the two *Lactobacillus* strains contributed to improved behavioral outcomes, we conducted the elevated plus maze (EPM) and open field test (OFT), classical models of anxiety-like behavior in rodents (Fig. 2A).

In the EPM, the combination treatment group exhibited a greater tendency for increased distance traveled in and time spent in the open arms compared to zymosan-injected mice on day 6 (Fig. S1B-S1D). This trend became statistically significant on day 13, with the combination treatment group showing markedly greater open arm exploration compared to other zymosan-induced groups, including the AMT treated group (Fig. 2B-2D).

Similarly, in the open field test (OFT) on day 7, zymosan-injected mice exhibited fewer entries into the central area, reduced distance traveled, and spent less time in the center compared to the control group (Fig. S1E-S1H). Although no significant differences were observed among the treatment groups at this early time point, the combination group display progressive behavioral improvements over time. By day 14, mice receiving the combined *Lactobacillus* this treatment showed significantly higher central entries, increased distance traveled, and longer time spent in the center relative to the zymosan control group, with effects that were more pronounced than those observed in other zymosan-induced groups (Fig. 2E-2G). These results demonstrate that combined administration of *L. paracasei* KBL382 and *L. plantarum* KBL396 effectively alleviates anxiety-like behaviors in a zymosan-induced IBS model.

### *Lactobacillus* Combination Treatment Ameliorates IBS-Like Symptoms Independent of Microbiota Dysbiosis

**Fig. 3:**
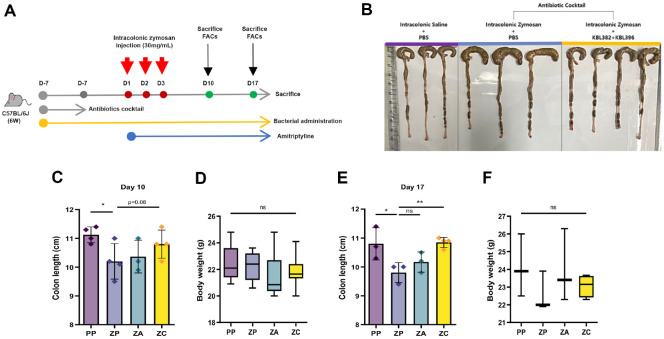
*Lactobacillus* combination treatment ameliorates IBS-like symptoms independently of antibiotic pre-treatment in a zymosan-induced mouse model. (**A**) Schematic representation of the zymosan-induced IBS model in SPF mice for in vivo studies. Mice were provided antibiotic-containing water for one week, followed by autoclaved tap water for the remainder of the experiment. The animals were orally administered a *Lactobacillus* mixture daily and colonized with the strains for 7 days before receiving intracolonic injection of zymosan suspension for three consecutive days, with administration continuing until the end of the experiment. (**B**) Representative photographs of the cecum and large intestine from each group at day 10. (**C** and **E**) Colon length measured at the time of euthanasia on days of sacrifice. (**D** and **F**) Body weight of PP, ZP, ZA, and ZC groups on the days of sacrifice. The box and horizontal bar in (**D** and **F**) represent the interquartile range and median of the correlation coefficients, respectively, while whiskers indicate the most extreme data points. Statistical analysis was performed using Mann-Whitney U-test. *P*-values are indicated as follows: *, *P* < 0.05; **, *P* < 0.01; ***, *P* < 0.001; ****, *P* < 0.0001; n.s. not significant. PP, mice receiving intracolonic injection of PBS and oral administration of PBS; ZP, mice receiving intracolonic injection of zymosan and oral administration of PBS; ZA, mice receiving intracolonic injection of zymosan and oral administration of amitriptyline; ZC, mice receiving intracolonic injection of zymosan and oral administration of *L. paracasei* KBL382 and *L. plantarum* KBL396 in combination.

Next, we questioned whether the therapeutic efficacy of the *Lactobacillus* combination depends on microbiota-mediated interactions by assessing its effects in zymosan-induced IBS mice with or without antibiotic pre-treatment (Fig. 3A). As previously observed in the non-antibiotic-treated, zymosan injection alone led to marked colon shortening and cecal shrinkage by day 7, whereas treatment with either the *Lactobacillus* combination or AMT restored both parameters (Fig. S2A and S2B). By day 14, colonic length and cecal size had normalized in all groups (Fig. S2C and S2D), consistent with the natural recovery of zymosan-induced IBS symptoms observed in mice with naive microbiota (Fig. 1B and 1C). These findings suggest that IBS symptoms can recover naturally in the presence of a healthy microbiota.

To further test the microbiota effects on zymosan induced IBS, a cohort was pretreated with a broad-spectrum antibiotic cocktail to deplete resident microbes, based on prior findings indicating that antibiotics induce microbiota imbalance or dysbiosis [33-35]. In these antibiotic-treated mice, zymosan injection induced abnormal cecal enlargement, which was notably absent in the combination treatment group (Fig. 3B). Zymosan also led to significant colon shortening on days 10 and 17, but this was reversed by the combination treatment by day 17 (Fig. 3C and 3E). No significant differences in body weight were observed among groups (Fig. 3D and 3F). Under antibiotic-induced gut microbiota dysbiosis, unlike the antibiotic-naïve group, untreated mice exhibited colon shortening induced by zymosan persisting up to day 17 (Fig. 3E). Notably, it was confirmed that the zymosan induced colon shortening effect persisting up to day 17 was significantly alleviated by the administration of *lactobacillus* combination (Fig. 3E).

### Combination Treatment with Two *Lactobacillus* Strains Suppresses Proinflammatory Immune Responses and Enhances Treg-Associated Immune Regulation

**Fig. 4:**
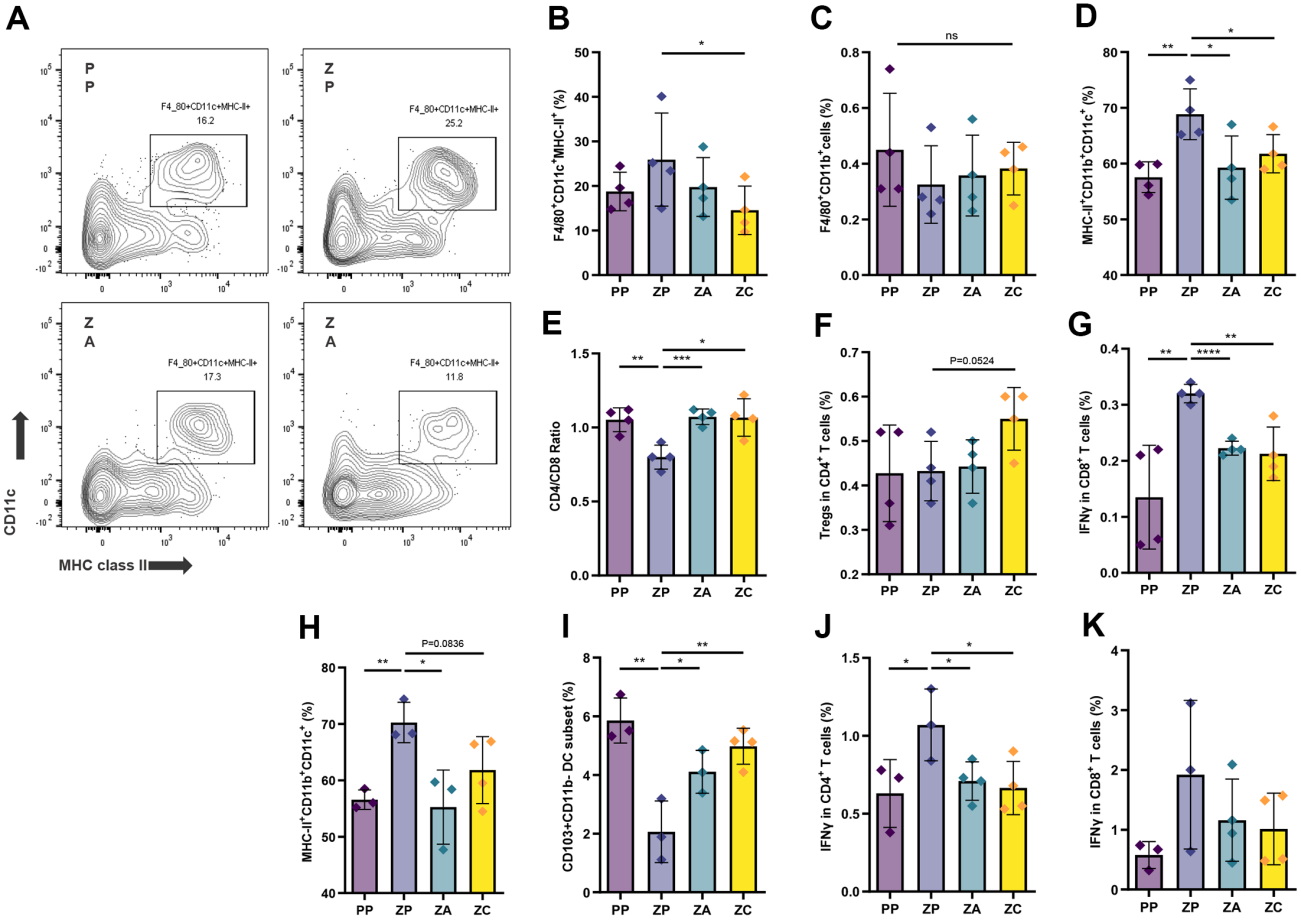
Combination treatment with two *Lactobacillus* species suppresses proinflammatory immune responses and enhances Treg activity. (**A**) Flow cytometry analysis of M1 macrophage levels using CD11c and MHC class II markers in CD11b^+^F4/80^+^ macrophage cells isolated from mesenteric lymph nodes following intracolonic injection and oral treatment with the indicated interventions. (**B** to **G**) Flow cytometry analysis of antigen-presenting cells (APCs; **B-D**) and T cells (**E-G**) isolated from mesenteric lymph nodes on day 10. (**H** to **K**) Flow cytometry analysis of APCs (**H** and **I**) and T cells (**J** and **K**) isolated from mesenteric lymph nodes on day 17. Symbols represent individual mice. The box and horizontal bar in panels (**B** and **K**) represent the interquartile range and median of the correlation coefficients, respectively, while whiskers indicate the most extreme data points. Statistical analysis was performed using Mann-Whitney U test. *P*-values are indicated as follows: *, *P* < 0.05; **, *P* < 0.01; ***, *P* < 0.001; ****, *P* < 0.0001; n.s. not significant.

The gut microbiota plays a central role in immune regulation and is tightly linked to brain function via the microbiota–gut–brain axis. To assess whether reduced colonic inflammation was associated with changes in immune cell composition, we performed flow cytometric profiling of immune subsets within the mesenteric lymph nodes (mLNs). Mice treated with the *Lactobacillus* combination showed a significant reduction in CD11c^+^MHC-II^+^CD11b^+^F4/80^+^ M1 macrophages compared to zymosan-injected controls (Fig. 4A-4C), while other macrophage subsets remained unchanged. Inflammatory dendritic cells (DCs) were also significantly reduced in the combination group, in contrast to the elevated levels observed in the zymosan group on day 10 (Fig. 4D).

Zymosan injection also decreased the CD4^+^:CD8^+^ T cell ratio (Fig. 4E and Fig. S3A). Although total CD4^+^ regulatory T cells (Tregs) frequencies were not significantly altered, the combination group showed a trend toward increased Treg abundance (Fig. 4F and Fig. S3B). The treatment also attenuated pro-inflammatory T cell activation. IFN-γ-producing CD8^+^ T cells were elevated in zymosan-injected mice but significantly reduced in both the combination and AMT-treated groups (Fig. 4G and Fig. S3C).

By day 17, inflammatory DCs remained elevated in the zymosan group, while the combination group showed a significant increase in CD103^+^CD11b^-^CD11c^+^ Treg-inducing DCs (Fig. 4H, 4I and Fig. S3D, S3E). IFN-γ-producing CD4^+^ T cells were also significantly decreased following combination treatment (4J and Fig. S3F), and a similar trend was observed for IFN-γ+ CD8^+^ T cells, although this did not reach statistical significance (Fig. 4K and Fig. S3G).

These results demonstrate that *Lactobacillus* combination therapy reprograms the immune landscape by suppressing pro-inflammatory subsets, including M1 macrophages, inflammatory DCs, and IFN-γ-producing T cells, while promoting Treg-inducing DCs. This shift toward a more tolerogenic immune profile likely contributes to both the reduction in intestinal inflammation and the alleviation of anxiety-like behaviors. Together, these findings underscore the potential of microbial immunomodulation as a therapeutic avenue for managing the multifactorial symptoms of IBS.

### Identification and Characterization of Bioactive Compartment in *Lactobacillus* Strains

To elucidate the bioactive compounds responsible for the immunomodulatory and anxiolytic effects of the *L. paracasei* KBL382, *L. plantarum* KBL396 in an IBS mouse model, we employed a stepwise fractionation and characterization strategy.

**Fig. 5:**
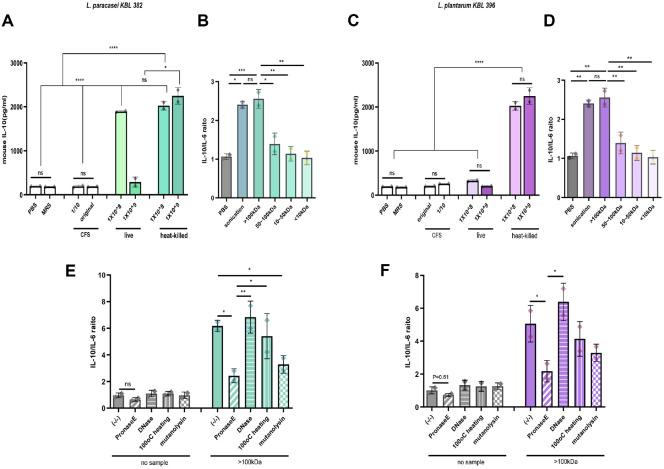
High-molecular-weight proteins and cell wall components from *L. paracasei* and *L. plantarum* enhance the IL-10/IL-6 ratio. (**A** and **B**) IL-10 levels produced by mouse splenocytes were measured after treatment with *L. paracasei* KBL382 (**A**) and *L. plantarum* KBL396 (**B**) using ELISA. Splenocytes were treated with PBS and cell-free supernatant as negative controls or with live or pasteurized bacteria and cultured for 24 h. (**C** and **D**) IL-10/IL-6 ratio in mouse splenocytes treated with various size fractions of *L. paracasei* KBL382 (**C**) and *L. plantarum* KBL396 (**D**). Samples were treated with a uniform protein concentration of 50 μg, standardized based on the BCA assay, and cultured for 6 h. (**E** and **F**) IL-10/IL-6 ratio in mouse splenocytes treated with compounds larger than 100 kDa from *L. paracasei* KBL382 (**E**) and *L. plantarum* KBL396 (**F**) for 24 h. The compounds were degraded using DNase, RNase, protease, and mutanolysin to remove effective molecules. To induce inflammation, 100 ng of LPS was added to all wells. PBS and each enzyme were used as controls. Supernatants were collected, and cytokine concentrations were measured using ELISA. Data are presented as mean ± SEM from three independent experiments. Statistical analysis was performed using one-way ANOVA followed by Tukey’s test. *P*-values are indicated as follows: *, *P* < 0.05; **, *P* < 0.01; ***, *P* < 0.001; ****, *P* < 0.0001; n.s., not significant. Symbols represent individual mice.

Live cells, heat-killed cells, and cell-free supernatants (CFS) from both strains were evaluated for their effects on LPS-stimulated mouse splenocytes at two concentrations. Heat-killed cells significantly increased IL-10 levels, whereas live *L. paracasei* KBL382 induced a dose-dependent reduction in IL-10 production, while no significant decrease was observed with *L. plantarum* KBL396 (Fig. 5A and 5C). Notably, CFS had no measurable effect, implying that non-secreted or structural components, rather than soluble metabolites, are likely responsible.

To further define the active components, bacterial cells were disrupted by sonication to release intracellular and structural elements while preserving heat-sensitive molecules. Lysates then were fractionated by ultrafiltration into four molecular weight ranges (>100 kDa, 50–100 kDa, 10–50 kDa, and < 10 kDa) and tested for immunomodulatory activity in LPS-stimulated splenocytes. The >100 kDa fraction most strongly increased the IL-10/IL-6 ratio, closely resembling the activity of whole sonicated lysates (Fig. 5B and 5D). However, differences were observed compared to pasteurized bacteria (Fig. S4A and S4B), possibly due to discrepancies in bioactive molecule abundance, as all samples were normalized by cell count rather than total protein content or biomass. This suggests that per-cell release of bioactive components differs among treatment modalities, influencing immune outcomes.

To characterize the nature of the bioactive macromolecules in the >100 kDa fraction, we applied enzymatic and thermal treatments. Pronase digestion significantly reduced IL-10/IL-6 ratios in both strains (Fig. 5E and 5F), and SDS-PAGE confirmed complete degradation (Fig. S4C), implicating proteins as primary mediators. Mutanolysin, a muramidase targeting the β-1,4-glycosidic bonds within the peptidoglycan backbone, also significantly diminished activity, particularly for *L. paracasei* KBL382. This suggests that intact peptidoglycan structures or associated complexes contribute functionally to immune activation, either independently or in synergy with protein components. DNase treatment had no effect, excluding a role for DNA. Heat treatment at 100°C resulted in a modest, non-significant reduction in activity (Fig. 5E and 5F), pointing to the possible involvement of heat-stable entities such as polysaccharides or protein–peptidoglycan complexes.

These findings identify high-molecular-weight proteins as key drivers of the observed anti-inflammatory effects. However, structurally integrated cofactors, likely peptidoglycan–protein complexes or other cell wall-associated elements, also appear to contribute to full immunostimulatory activity. These data underscore the importance of multicomponent interactions in shaping the host immune response to *Lactobacillus* strains.

### Effective Proteins in >100 kDa Fractions of *Lactobacillus* spp. Modulate TLR Signaling and Upregulate SERT1 Expression

**Fig. 6:**
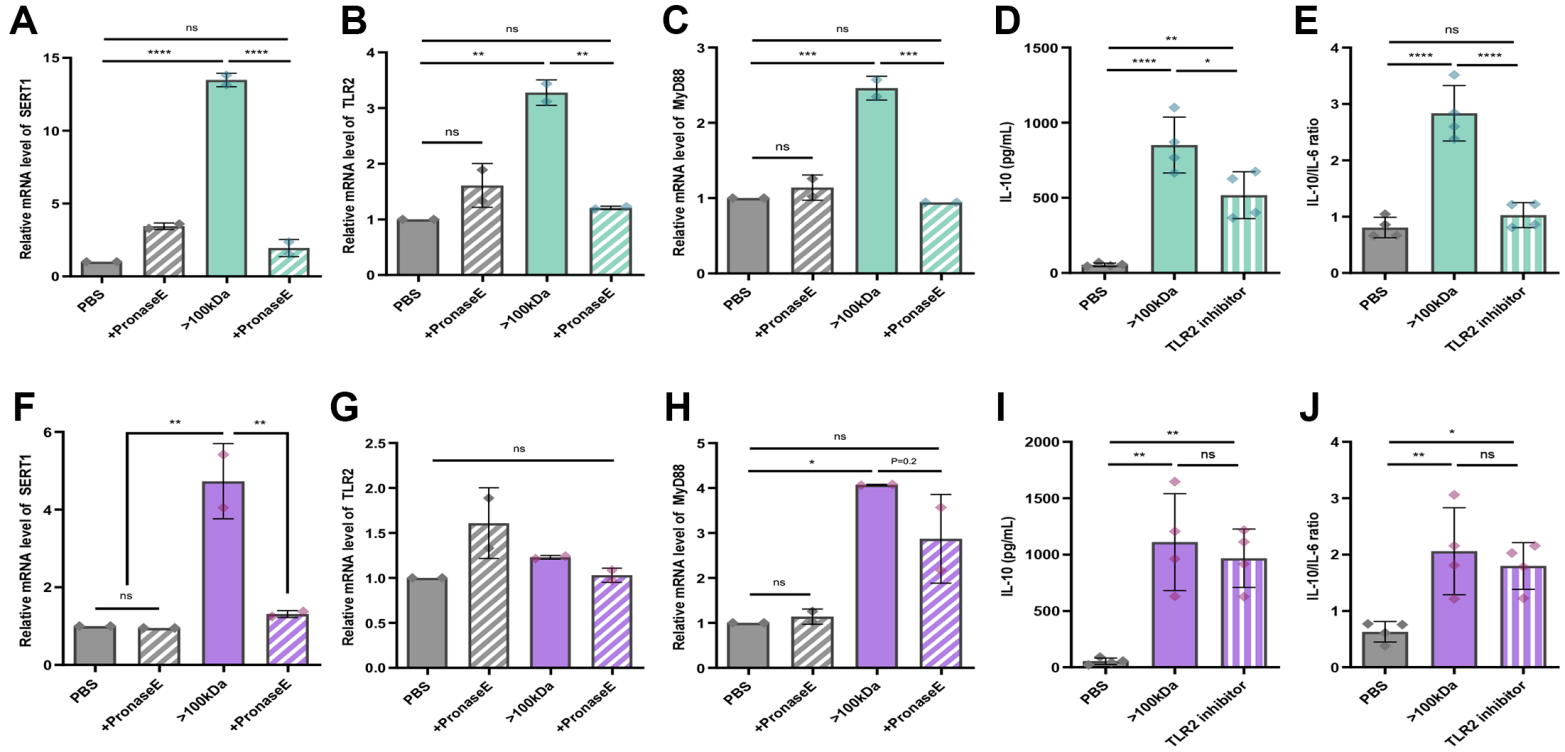
Proteins in >100 kDa fractions of *Lactobacillus* spp. enhance TLR2 and MyD88 expression and upregulate SERT. (**A** to **C**) Quantification of SERT (**A**), TLR2 (**B**), and MyD88 (**C**) expression in mouse splenocytes treated for 24 h with pasteurized *L. paracasei* KBL382 (with or without proteins, as determined by protease treatment) or >100 kDa fractions (with or without proteins), measured by real-time qPCR. (**D**) IL-10 levels in splenocytes treated with >100 kDa fractions of *L. paracasei* KBL382, measured by ELISA. (**E**) IL-10/IL-6 ratio in splenocytes treated with or without a TLR2 inhibitor for 24 h. (**F** to **H**) Quantification of SERT1 (**F**), TLR2 (**G**), and MyD88 (**H**) expression in splenocytes treated with pasteurized *L. plantarum* KBL396 (with or without proteins) or >100 kDa *L. plantarum* KBL396 fractions (with or without proteins, as determined by protease treatment) for 24 h, measured by real-time qPCR. (**I**) IL-10 levels in splenocytes treated with >100 kDa fractions of *L. plantarum* KBL396 measured by ELISA. (**J**) IL-10/IL-6 ratio in splenocytes were treated with or without TLR2 inhibitor for 24 h. PBS served as a negative control, while enzyme-treated PBS was used as a blank. To induce inflammation, LPS (100 ng/ml) was added to all wells. Supernatants were collected, and cytokine concentrations were determined by ELISA. Data are presented as mean ± SEM of three independent experiments. Statistical analysis was performed using one-way ANOVA followed by Tukey’s test. *P*-values. *P*-values are indicated as follows: *, *P* < 0.05; **, *P* < 0.01; ***, *P* < 0.001; ****, *P* < 0.0001; n.s., not significant.

To assess whether the *in vivo* upregulation of SERT1 was mediated by high-molecular-weight bacterial components, we conducted in vitro experiments using LPS-stimulated mouse splenocytes. Treatment with pasteurized *Lactobacillus* strains, either individually or in combination, significantly increased SERT1 expression relative to PBS controls, with the combination treatment eliciting a more pronounced effect than either strain alone (Fig. S4D). Similarly, exposure to >100 kDa fractions from both strains led to a significant increase in SERT1 expression, which was completely abolished by protease treatment (Fig. 6A and 6F). These findings indicate that proteinaceous components within the high-molecular-weight fractions are required for modulating SERT1, suggesting a mechanism by which these strains influence gut-brain axis signaling.

In parallel, we investigated whether the same >100 kDa fractions modulate innate immune signaling, based on the immune cell phenotypes observed following combination treatment with *L. paracasei* KBL382 and *L. plantarum* KBL396 *in vivo*.

Innate immune signaling in the gut is primarily regulated by pattern recognition receptors (PRRs), such as Toll-like receptors (TLRs) and NOD-like receptors (NLRs), which serve as key initiators of cytokine production [28, 29]. To investigate how *Lactobacillus*-derived macromolecules engage these pathways, we analyzed PRR signaling in response to the >100 kDa fractions. The anti-inflammatory activity was significantly reduced following pronase treatment, indicating that proteins are the primary bioactive components. Given this result, we focused on TLR2, which is known to recognize microbial proteins and lipoproteins, as a potential target receptor [36, 37]. In *L. paracasei* KBL382, TLR2 expression was significantly upregulated following treatment, whereas *L. plantarum* KBL396 did not induce a similar change (Fig. 6B and 6G). Notably, the increase in TLR2 expression by *L. paracasei* KBL382 was abolished by protease treatment, indicating that protein components are essential for TLR2 engagement. In line with this, MyD88, an adaptor protein involved in TLR signaling, was upregulated in response to the >100 kDa fractions from both strains, and this response was similarly eliminated by protease digestion (Fig. 6C and 6H), further supporting the central role of protein mediators.

To further confirm the involvement of TLR2 in mediating the effects of high-molecular-weight bacterial proteins, we performed inhibition experiments using a selective TLR2 antagonist. In splenocytes treated with >100 kDa fractions of *L. paracasei* KBL382, IL-10 secretion and the IL-10/IL-6 ratio were significantly increased compared with PBS controls (Fig. 6D and 6E). These anti-inflammatory effects were largely abolished in the presence of the TLR2 inhibitor, demonstrating that the immunoregulatory activity of *L. paracasei* KBL382- derived protein fractions is critically dependent on TLR2 signaling. By contrast, the corresponding fractions from *L. plantarum* KBL396 did not elicit significant TLR2-mediated cytokine modulation, and the inhibitory effect was less evident (Fig. 6I and 6J). These results provide functional evidence that TLR2 engagement underlies the immunomodulatory activity of *L. paracasei* KBL382, thereby strengthening the mechanistic link between strainspecific protein complexes and host innate immune signaling.

Together, these results highlight the dual immunomodulatory and neuroregulatory potential of high-molecular-weight protein complexes derived from *Lactobacillus* strains. They suggest mechanistic pathways through which these macromolecular assemblies contribute to the therapeutic effects observed in IBS.

## Discussion

The gut microbiota plays a central role in regulating immune responses and neurobehavioral functions through the gut microbiota–immune–brain axis. However, the mechanisms underlying this complex communication network remain incompletely understood. In this study, we provide compelling evidence that a combination of *L. paracasei* KBL382 and *L. plantarum* KBL396 exerts synergistic therapeutic effects in a zymosan-induced mouse model of IBS. The treatment significantly alleviated IBS-like symptoms, including shortened colon length, abnormal cecal morphology, inflammation, and anxiety-like behavior. These effects were accompanied by bidirectional modulation of neurobiological markers in the gut and brain, with reduced expression of colonic BDNF and 5-HT3A receptors, both of which are implicated in visceral hypersensitivity, and increased levels of hippocampal BDNF and serotonin receptors, which are associated with mood improvement. The dual action of the combined treatment on both intestinal and central pathways underscores its potential as a multifaceted therapeutic approach for addressing both the gastrointestinal and psychological dimensions of IBS.

At the cellular level, the combination treatment markedly reshaped the immune landscape of mesenteric lymph nodes. It significantly reduced the abundance of pro-inflammatory M1 macrophages and inflammatory dendritic cells (DCs), while increasing the population of CD103^+^CD11b^-^ DCs, a subset known to induce regulatory T cells (Tregs) in the gut-associated lymphoid tissue [38]. Correspondingly, there was an enhancement of CD4^+^ Tregs, which play a critical role in maintaining immune tolerance, suppressing excessive inflammatory responses, and preserving intestinal homeostasis. These immunological shifts are particularly relevant in the context of the gut microbiota–immune–brain axis, a bidirectional communication network wherein microbial signals modulate both local immune responses and central nervous system function. Mounting evidence indicates that gut-derived immune signals contribute to the pathophysiology of psychiatric conditions, including anxiety and depression [39-41]. Elevated levels of pro-inflammatory cytokines such as IL-6, TNF-α, and C-reactive protein (CRP) are commonly observed in individuals with inflammatory depression and have been linked to increased vulnerability to mood disorders [42, 43]. Moreover, experimental induction of systemic inflammation has been shown to provoke anxiety-like behaviors in both humans and animal models [44, 45]. These data underscore the pathophysiological significance of chronic low-grade inflammation in both intestinal and neuropsychiatric manifestations of IBS. Thus, the observed immunomodulatory effects of the *Lactobacillus* combination not only alleviate intestinal inflammation but may also attenuate neuroinflammatory signaling pathways, reinforcing the therapeutic relevance of targeting the immune system to address the multifactorial nature of IBS.

Conventional treatments for IBS, including tricyclic antidepressants (*e.g.*, amitriptyline), serotonin receptor modulators, selective serotonin reuptake inhibitors (SSRIs), and non-absorbable antibiotics such as rifaximin, provide partial symptom relief but are often limited by side adverse effects and fail to address the full range of gastrointestinal and neuropsychological manifestations. In contrast, the combination of *L. paracasei* KBL382 and *L. plantarum* KBL396 demonstrated superior efficacy compared to amitriptyline in our study, without inducing systemic toxicity or weight loss. Unlike single-target pharmacologic agents, this probiotic combination concurrently modulated mucosal inflammation and central neurobiological pathways, thereby targeting both peripheral and central components of IBS pathophysiology. These findings support its potential as a safe and effective microbiome-based therapeutic strategy for comprehensive IBS management.

Interestingly, high-dose amitriptyline administration in mice led to significant weight loss, which contrasts with the weight gain commonly reported as a side effect in humans [46]. This discrepancy may reflect interspecies pharmacokinetic differences and systemic effects of high-dose treatment that influence feeding behavior and energy metabolism. In humans, weight gain is typically associated with chronic administration, whereas our experimental design involved short-term, high-dose exposure that may trigger distinct mechanisms of body weight regulation. Consistent with this notion, chronic TCA treatment has not been shown to increase daily food intake or promote body weight gain; rather, desipramine consistently caused decreased food intake and weight loss, while amitriptyline either had no effect or slightly reduced body weight compared to controls [47]. Furthermore, previous reports demonstrate that the effects of amitriptyline are dose-dependent, with low doses eliciting specific behavioral or physiological responses, but high doses producing toxic effects that can result in markedly different outcomes [48]. Clinically, these findings underscore the importance of dose optimization, as treatment-induced weight changes may impact patient tolerability and adherence.

Another key observation is that the therapeutic effects of the *Lactobacillus* combination were maintained even in microbiota-imbalanced mice. Prolonged restoration of colon morphology and improvement in behavioral symptoms occurred despite the dysbiosis of native microbial communities, whereas the existing IBS drug (AMT) showed no therapeutics effects. These findings suggest that IBS occurring with gut microbiota dysbiosis may lead to worsened or prolonged symptoms, which could be partly attributed to the residual commensal microbe remaining after antibiotic treatment and may be related to the ineffectiveness of existing therapy. In contrast, the *Lactobacillus* combination demonstrated potential as an effective therapeutic approach against microbiota dysbiosis associated IBS. The consistency of these effects across microbial contexts highlights the robustness and translational potential of this postbiotic strategy for the treatment of IBS and its associated neurobehavioral symptoms.

A central mechanistic finding of our study is the identification of high-molecular-weight (>100 kDa) bioactive macromolecular complexes enriched in PGN–protein structures, which were responsible for both immuno-modulatory and neuroregulatory effects. These fractions increased the IL-10/IL-6 ratio and upregulated SERT1 expression in vitro. Enzymatic degradation of protein components or digestion of PGN by mutanolysin abolished these effects, indicating that the structural integrity of PGN–protein complexes is essential for their biological activity. In vitro ELISA showed the most significant change following treatment with pronase, which degrade proteins. This data suggests that specific proteins within macromolecular fraction may act as key immuno-modulatory molecule. In future study, plan to perform proteomic analyses to identify the protein responsible for the observed effects. Such identification will be essential for elucidating the precise molecular mechanisms and for guiding the development of targeted therapeutic applications.

Previous studies have shown that bacterial proteins, such as Loa22 from *Leptospira*, can enhance PGN-mediated activation of TLR2, promoting downstream immune responses [49, 50]. Consistent with this, our data demonstrate that these complexes engage the TLR2–MyD88 signaling axis, a pathway known to promote IL-10 production and regulatory T cell differentiation [51-53]. Inhibitor experiments further confirmed that the immunomodulatory activity of *L. paracasei* KBL382-derived proteins is critically dependent on TLR2, whereas *L. plantarum* KBL396 did not show a similar reliance, highlighting strain-specific engagement of innate immune pathways. These findings support a model in which structurally integrated PGN–protein assemblies are required not only for innate immune activation but also for modulation of serotonergic signaling along the gut–brain axis.

While both strains converge on MyD88-dependent innate immune pathways, *L. plantarum* KBL396 upregulated MyD88 expression without a significant change in TLR2 levels. This differential activation profile suggests that the two strains may engage partially overlapping yet distinct PRR pathways, potentially contributing to their synergistic immunomodulatory and neuroregulatory effects when administered in combination. Despite these mechanistic insights, further studies are needed to identify the specific upstream receptor involved in *L. plantarum* KBL396-mediated signaling and to clarify whether differential PRR engagement underlies the enhanced therapeutic efficacy observed with the combined treatment.

Furthermore, we found that treatment with live *Lactobacillus* strains induced a dose-dependent immune response, wherein higher bacterial loads led to a significant reduction in IL-10 production. These findings are consistent with prior reports that *Lactobacillus* species exhibit context-dependent immunomodulatory effects, shifting from anti-inflammatory to pro-inflammatory activity under certain pathological conditions [54]. In this study, the zymosan-induced model was employed because it recapitulates key features of IBS, including visceral hypersensitivity, altered gut motility, and persistent low-grade inflammation in the absence of structural damage. Compared with models such as TNBS colitis or water avoidance stress, the zymosan model provides a more translationally relevant phenotype for functional bowel disorders [28-32]. Nevertheless, it is important to acknowledge that this model predominately reflects IBS-D-like phenotypes. Future investigations should therefore validate these findings across other IBS subtypes to ensure broader applicability.

Although rodent anxiety-like behavioral tests provide useful insights into gut–brain interactions, their translational relevance to human IBS-related psychological conditions remains limited. Human IBS patients experience complex psychological manifestations, including stress, anxiety, and altered coping behaviors, which cannot be fully recapitulated in rodent models. Therefore, findings from rodent anxiety paradigms should be interpreted with caution, and complementary approaches in clinical or translational settings are needed to bridge this gap.

In conclusion, this study presents a robust and multifaceted mechanism through which a defined combination of *Lactobacillus* strains exerts immune and neurobiological regulation in an IBS context. By identifying structurally integrated PGN–protein complexes as the key mediators, we provide foundational insights for the development of next-generation postbiotics. These formulations could bypass the safety concerns of live bacteria while delivering targeted therapeutic benefits. Further studies are warranted to establish their efficacy across diverse IBS subtypes and ultimately translate these findings into clinically viable microbiome-based interventions.

## Supplemental Materials

Supplementary data for this paper are available on-line only at http://jmb.or.kr.
