# Supplementary material for: Synergistic Modulation of the Gut–Brain–Immune Axis by a Dual Lactobacillus Combination in a Murine IBS Model
Source: J Microbiol Biotechnol. 2025 Oct 27;35:e2507018. doi: 10.4014/jmb.2507.07018 (PMC12603378; doi:10.4014/jmb.2507.07018)
Supplement: Supplementary file 1 [file jmb-35-e2507018-supple.pdf]

**J. Microbiol. Biotechnol. 2025. 35: e2507018**
**https://doi.org/10.4014/jmb.2507.07018**

# Supplemental Methods

## Bacteria Preparation

For the in vivo experiment, The *Lactobacillus* strains were lyophilized individually for administration to mice. Prior to administration, each lyophilized strain was diluted 10-fold, and colony counts were determined using the pour plate method to assess viability. The lyophilized bacteria were rehydrated in phosphate-buffered saline (PBS) at a concentration of $10^9$ colony-forming units (CFU) per milliliter. The bacterial solution was administered orally to mice using a plastic feeding needle at consistent times each day for three weeks, beginning seven days prior to zymosan injection and continuing throughout the experimental procedure until the mice were sacrificed. For the in vitro experiment, the bacterial strains were revived at a 1:100 dilution and cultured in MRS broth (KisanBio, Seoul, Republic of Korea) for 9 hours, followed by subculturing for an additional 13 hours. After incubation, the cultures were centrifuged at 5,000 × g for 20 min at 4°C. The resulting bacterial pellets were washed with PBS, centrifuged again, and resuspended in PBS. Bacterial cell counts were determined using an Accuri C6 Flow Cytometer (BD Biosciences, USA) and the LIVE/DEAD™ BacLight™ Bacterial Viability and Counting Kit (Invitrogen, USA). For pasteurization, the bacteria were heat-treated at 65°C for 30 min.

## Bacterial fractionation

Pasteurized bacteria were sonicated using a VC505 Ultrasonic Processor (Sonics, USA) for 1 hour at 30% amplitude with 15-sec pulses followed by 45-sec pauses (15s on/45s off). The samples were kept on ice throughout the sonication process to prevent protein degradation. Sonicated bacteria were centrifuged at 10,000 × g for 20 min at 4°C. The resulting pellet (cell debris) was resuspended in PBS, while the supernatant (cell lysate) was further centrifuged using an Optima™ L-100 XP Ultracentrifuge (Beckman, USA) at 100,000 × g for 1 h at 4°C. The pellet (crude membrane fraction) was resuspended in PBS. All fractions were stored at −20°C until further use. For size-based fractionation, the sonicated bacteria were separated using Amicon® Ultra Centrifugal Filters (Sigma-Aldrich, USA) into fractions of >100 kDa,

50–100 kDa, and 10–50 kDa. To remove DNA, proteins, and peptidoglycan from pasteurized bacteria, sonicated bacteria, and bacterial fractions, samples were treated with DNase I (Sigma-Aldrich, USA), pronase E (Sigma-Aldrich), and mutanolysin. For protein degradation, samples were treated with pronase E (1 mg/mL) and incubated with shaking at 37°C for 12 h, followed by enzyme inactivation at 95°C for 5 min. DNA removal was performed using DNase I (100 μg/mL) with shaking incubation at 37°C for 60 min, and the enzyme was inactivated at 68°C for 10 min. RNA was removed by heating the samples at 100 °C for 15 min. Peptidoglycan degradation was achieved by treating the samples with mutanolysin (100 μg/mL) and incubating them overnight at 37°C, followed by enzyme inactivation at 100°C for 5 min. Blank controls were prepared by adding each enzyme to PBS and subjecting them to identical treatment protocols. Complete protein removal was confirmed via SDS-PAGE analysis of the pronase E-treated samples.

### Transcript analysis

Total RNA was extracted from mouse distal colon, hippocampus, and prefrontal cortex tissues using TRIzol reagent (Invitrogen, USA) and purified with the easy-spin™ Total RNA Extraction Kit (iNtRON Biotechnology, Republic of Korea), following the manufacturer's instructions. For Caco-2 cells and mouse splenocytes, total RNA was extracted using the easy-BLUE™ Total RNA Extraction Kit (iNtRON Biotechnology). RNA concentration and purity were measured using a NanoDrop ND-2000 spectrophotometer (Thermo Fisher Scientific, France). Complementary DNA (cDNA) was synthesized from total RNA using the High-Capacity RNA-to-cDNA Kit (Thermo Fisher Scientific, USA). Quantitative real-time PCR (qPCR) was performed using THUNDERBIRD™ Next SYBR® qPCR Mix (TOYOBO, Japan) on a QuantStudio 6 Flex Real-Time PCR System (Thermo Fisher Scientific, USA). Primer sequences used for amplification were as follows: mTNF-α: F 5′-CATCTTCTCAAAATTCGAGTGACAA-3′, R 5′-TGGGAGTAGACAAGGTACAACCC-3' mIL-1β: F 5′-GAAATGCCACCTTTTGACAGT-3′, R 5′-CTGGATGCTCTCATCAGGACA-3′ mBDNF: F 5′-GCGCCCATGAAAGAAGTAAA-3′, R 5′-TCGTCAGACCTCTCGAACCT-3′ mBDNF4: F 5′-CAGAGCAGCTGCCTTGATGTT-3′, R 5′-GCCTTGTCCGTGGACGTTTA-3′ mSERT1: F 5′-CTTCAGCCCCGGATGGTT-3′, R 5′-GTGGACTCATCAAAAAACTGCAAA-3′ m5HT3A: F 5′-AACAGCTATGCAGAAATGAAGTT-3′, R 5′-GGCTGACTGCGTAGAATAAAGG-3′ mTLR2: F 5′-TGGAATGTCACCAGGCTGC-3′, R 5′-GTCCGTGGAAATGGTGGC-3′

mMyD88: F 5′-ACCTGTGTCTGGTCCATTGCCA-3′, R 5′-GCTGAGTGCAAACTTGGTCTGG-3′ mGAPDH: F 5′-ATTGTCAGCAATGCATCCTG-3′, R 5′-ATGGACTGTGGTCATGAGCC-3′ hOCCLUDIN: F 5′-CCAATGTCGAGGAGTGGG-3′, R 5′-CGCTGCTGTAACGAGGCT-3′ hGAPDH: F 5′-CGGAGTCAACGGATTTGGTCGTAT-3′, R 5′-AGCCTTCTCCATGGTGGTGAAGAC-3′. GAPDH was used as the housekeeping gene for normalization. All reactions were performed in duplicate. Gene expression levels were calculated using the 2^–ΔΔCT method.

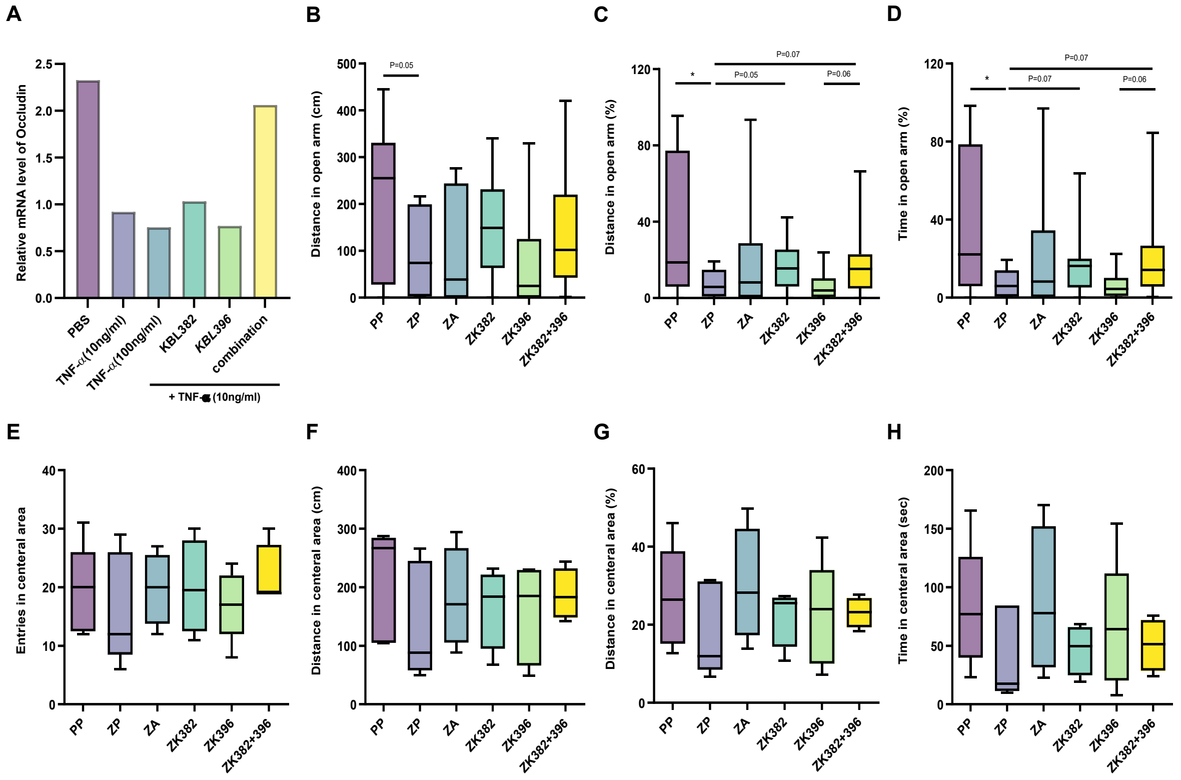


**Fig. S1. Bacterial administration attenuates zymosan-induced anxiety-like behavior.**

**(A)** Quantification of occludin expression caco-2 cells treated with heat-killed(pasteurized) *Lactobacillus* stains, both individually and in combination, for 48 h, as measured by real-time qPCR. (B to D) Summarized data of distance travel (**B and C**) and time spent **(D)** in the open arm of the EPM for the indicated groups on day 6. (**E to H**) Summarized data of entries (**E**), distance travel (**F and G**, and time spent(**H**) in the central area of the OFT from the indicated groups on day 7. Statistical analysis was performed using an unpaired two-tailed t-test. P-values are indicated as follows: *, $P < 0.05$; **, $P < 0.01$; ***, $P < 0.001$; ****, $P < 0.0001$; n.s. not significant.

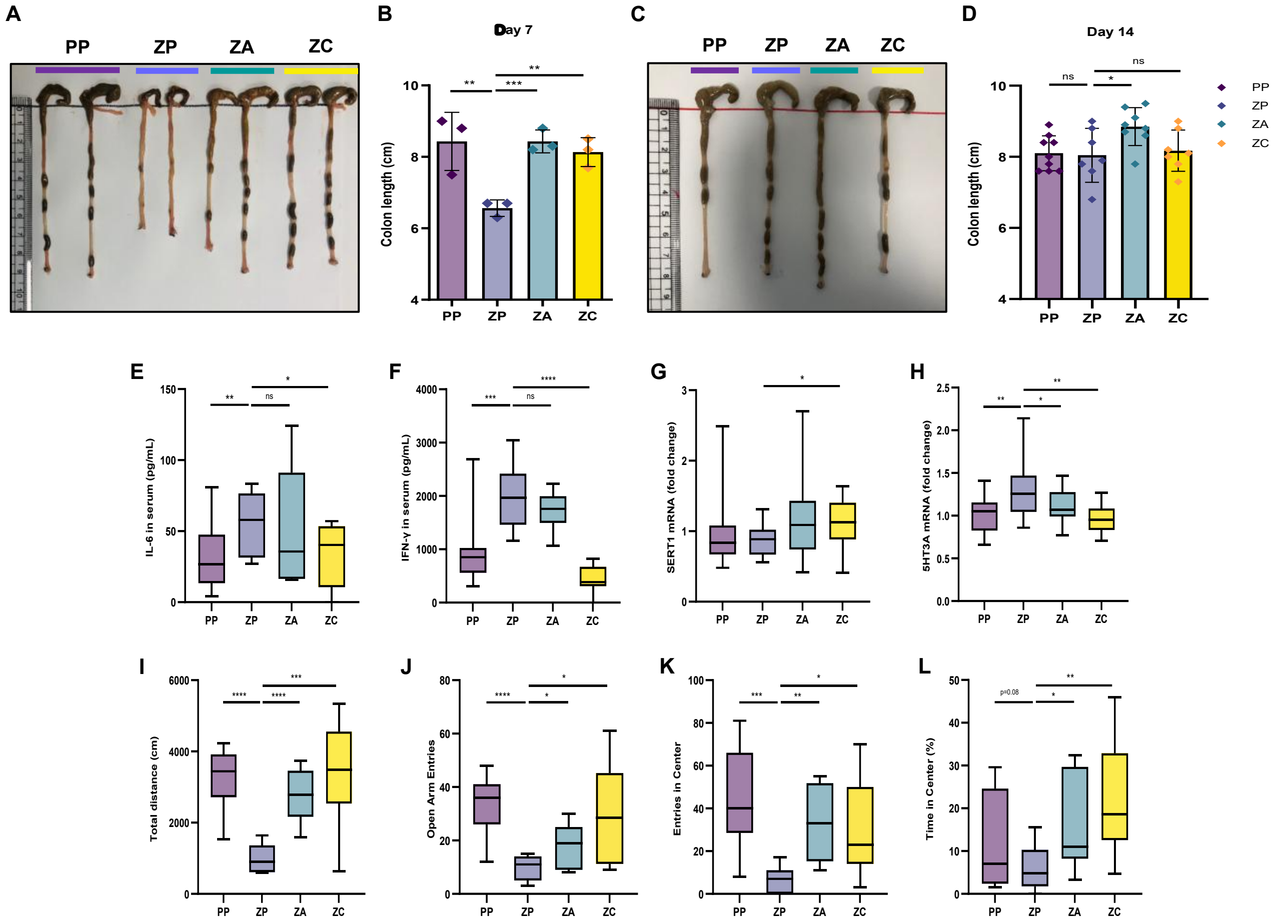


**Fig. S2. Combination treatment effect in a zymosan-induced murine model of IBS.**

Oral administration was started 7 days prior to zymosan injection and continued until the end of the experiment. Intracolonic injection of zymosan suspension was performed over three consecutive days. **(A and C)** Representative photographs of colon tissues from each group at days 4 and 14. **(B and D)** Colon length measured at the time of euthanasia on days 7 and 14. (**E and F**) Levels of IL-6 (**E**) and IFN-γ **(F)** in serum at day 14, determined by ELISA. (**G and H**) Quantification of SERT1 (**G**), and 5HT3A (**H**) expression in colon by real-time qPCR. Expression levels were normalized to GAPDH expression. (**I to L**) Anxiety-related behavior were determined by EPM test on day 6. Total distance (**I**), number of entries in open arm (**J**), number of entries in center (**K**), time spent in center (**L**) during the EPM test. The box and horizontal bar in (**E to L**) represent the interquartile range and median of the correlation coefficients, respectively, while whiskers indicate the most extreme data points. Statistical analysis was performed using an unpaired two-tailed t-test. P-values are indicated as follows: *, $P < 0.05$; **, $P < 0.01$; ***, $P < 0.001$; ****, $P < 0.0001$; n.s. not significant.

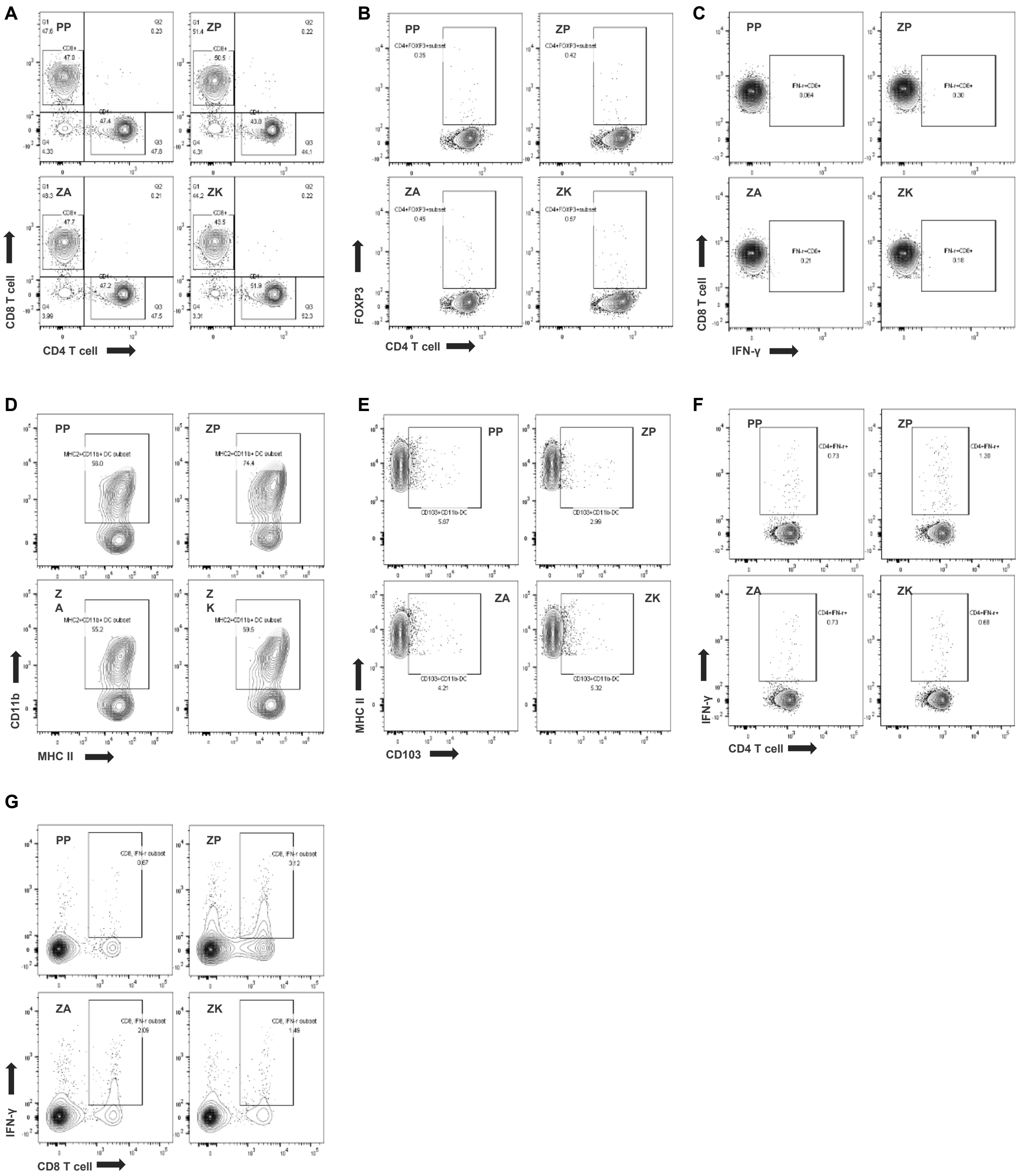


**Fig. S3. Flow cytometry gating strategy for analysis of T cell subsets and functional markers.**

Representative flow cytometry plots illustrating the gating strategy used for immune cell analysis. **(A)** Initial gating of CD4$^+$ and CD8$^+$ T cells from the CD3$^+$ population. **(B)** Identification of regulatory T cells (CD4$^+$Foxp3$^+$). (**C)** Detection of IFN-γ$^+$ CD8$^+$ T cells. **(D)** Gating of MHCII$^+$CD11b$^+$ dendritic cell subset. **(E)** Identification of CD101$^+$CD11b$^-$ dendritic cell subset. (**F**) Detection of IFN-γ$^+$ CD4$^+$ T cells. (**G)** Detection of IFN-γ$^+$ CD8$^+$ T cells. Data shown are representative of three independent experiments and demonstrate the sequential gating approach applied throughout the study.

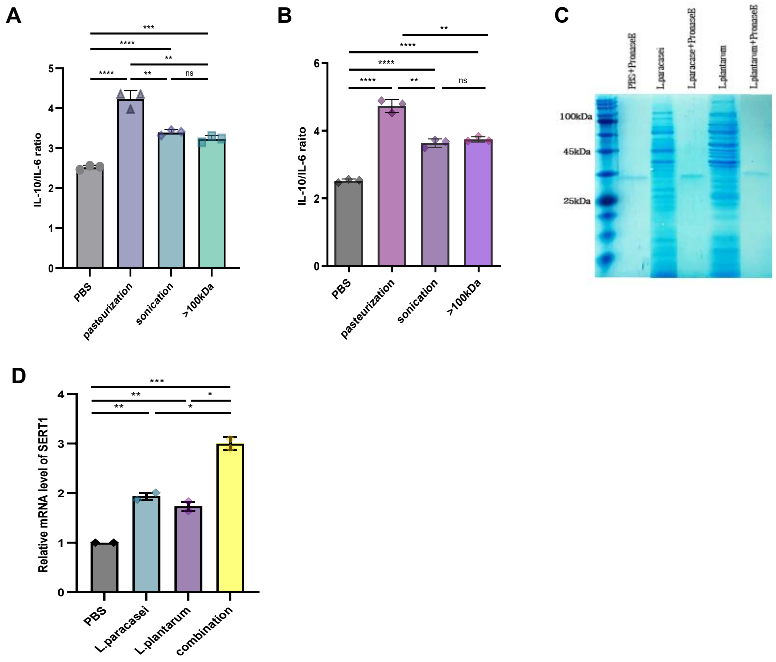


**Figure S4. Identification of bioactive compounds in *Lactobacillus* strains.**

**(A and B)** IL-10/IL-6 ratio in mouse splenocytes treated with pasteurized, sonicated, and >100 kDa fractions from *L. paracasei* (**A**) and *L. plantarum* (**B**). (**C**) SDS–PAGE analysis confirming the complete removal of proteins after ponase treatment. (**D**) Quantification of SERT1 expression in mouse splenocytes treated with single or combination of pasteurized *Lactobacillu*s strains for 24 h, as measured by real-time qPCR. To induce inflammation, 100 ng of LPS was added to all wells. PBS served as a negative control, while PBS treated with each enzyme was used as a blank. Supernatants were collected, and cytokine concentrations were measured using ELISA. Data are presented as the mean ± SEM from three independent experiments. Statistical analysis was performed using one-way ANOVA.P-values are indicated as follows: *, $P < 0.05$; **, $P < 0.01$; ***, $P < 0.001$; ****, $P < 0.0001$; n.s., not significant.
